# Risk factors of hyperkalemia after total parathyroidectomy in patients with secondary hyperparathyroidism

**DOI:** 10.1080/0886022X.2020.1803088

**Published:** 2020-10-07

**Authors:** Yun Zou, Liwei Zhang, Hua Zhou, Yan Yang, Min Yang, Jia Di

**Affiliations:** a Department of Nephrology, The Third Affiliated Hospital of Soochow University, Changzhou, China; b Department of Infection Management, The Third Affiliated Hospital of Soochow University, Changzhou, China

**Figure 1.**
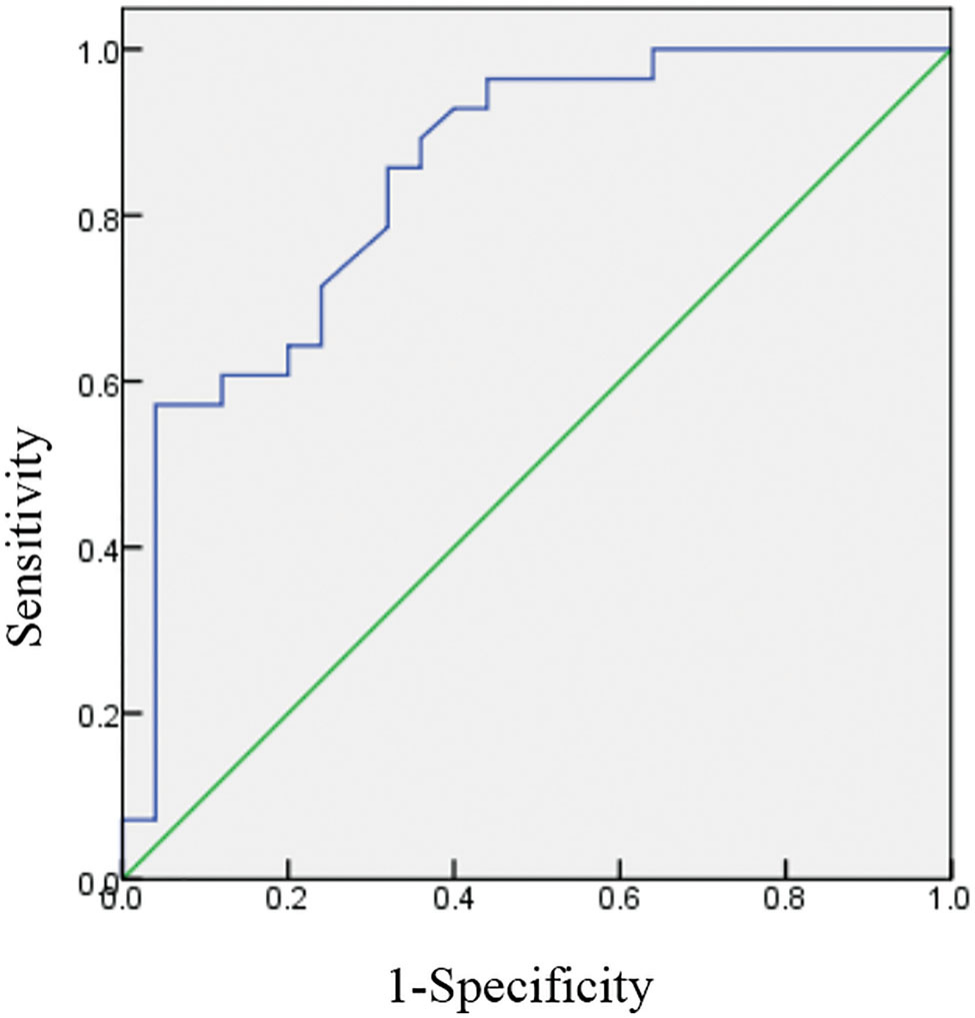
Receiver operating characteristic (ROC) curve of preoperative serum potassium associated with postoperative hyperkalemia.

Secondary hyperparathyroidism (SHPT) is a common complication in patients with end-stage renal disease (ESRD) and parathyroidectomy (PTX) is an effective treatment for SHPT. To examine the differential risk of post-surgical hyperkalemia after PTX for primary versus secondary hyperparathyroidism, we conducted a single-center retrospective observational study in 103 PTX patients admitted to the Third Affiliated Hospital of Soochow University between January, 2013 and August, 2019. Patients were divided into two groups according to pathogeny. PHPT group included symptomatic PHPT and asymptomatic PHPT with hypercalcemia. SHPT group included patients with maintenance hemodialysis for more than 3 months and having undergoing successful PTX. All SHPT patients received endoscopic total parathyroidectomy and forearm autotransplantation (tPTX + AT), while PHPT patients underwent open or endoscopic parathyroid adenomaectomy. Table 1 shows the clinical characteristics of patients. No patients in PHPT group were diagnosed as hyperkalemia after surgery, while postoperative serum potassium (K^+^_post_) was more than 5.3 mmol in 28 (52.8%) patients with SHPT. Then, SHPT patients were further divided into hyperkalemia group and nonhyperkalemia group according to K^+^_post_. Compared with nonhyperkalemia group, K^+^_base_ and K^+^_pre_ were significantly higher in hyperkalemia group (Table 2, *p* < 0.001). Preoperative iPTH and the decline range of iPTH in hyperkalemia group were higher than those in nonhyperkalemia group, but there was no statistical significance (*p* = 0.095, *p* = 0.066). There was no significant difference in age, gender, BMI, dialysis age, HCO3-, Hb, BUN, SCr, ALP, Chol, TG, UA, serum-corrected calcium, phosphorus, magnesium, ACEI/ARB, Cinacalcet between two groups (Table 2). We chose the variables with *p* < 0.1 for multivariate Logistic regression analysis. K^+^_pre_ was an independent influencing factor of postoperative hyperkalemia (OR = 18.888, 95%CI = 1.798–198.450, *p* = 0.014). ROC curve analysis showed that area under the curve (AUC) of K^+^_pre_ in predicting postoperative hyperkalemia was 0.844 (*p* < 0.001). The optimal cutoff value of K^+^_pre_ to predict hyperkalemia in SHPT patients after tPTX + AT was 4.30 mmol/L, with a sensitivity of 96.4% and a specificity of 56% (Figure 1).

**Table 1.** Comparison of perioperative clinical data of patients with SHPT and PHPT.

Characteristics	SHPT (*n* = 53)	PHPT (*n* = 50)	*p* value
Gender (*n*, male/female)	31/22	20/30	0.061
Age (year)	48.98 ± 12.39	53.70 ± 13.48	0.067
BMI (kg/m^2^)	21.41 ± 2.89	22.53 ± 3.43	0.075
Whether or not taking ACEI/ARB(*n*, yes/no)	7/46	5/45	0.612
preoperative iPTH (pg/ml)	2104.90 (1394.85,2739.00)	238.85 (130.93,485.78)	<0.001*
preoperative ALP (u/L)	453.00 (235.50, 930.00)	145.00 (113.50,214.00)	<0.001*
preoperative K^+^ (mmol/L)	4.60 (4.23, 4.98)	4.27 (3.79,4.50)	<0.001*
preoperative HCO3^-^ (mmol/L)	22.79 ± 2.80	23.02 ± 2.74	0.676
preoperative BUN (mmol/L)	15.56 (12.80,19.83)	4.41 (3.55,5.47)	<0.001*
preoperative Scr (umol/L)	700.00 (579.25,850.00)	73.50 (54.75,98.00)	<0.001*
preoperative UA (umol/L)	313.93 ± 89.89	328.55 ± 114.27	0.471
preoperative Alb (g/L)	37.36 ± 4.72	40.85 ± 5.04	<0.001*
preoperative P (mmol/L)	2.13 (2.34,1.94)	0.82 (0.64,0.96)	<0.001*
preoperative Mg (mmol/L)	1.07 (0.97,1.22)	0.93 (0.81, 1.06)	<0.001*
Chol (mmol/L)	3.82 (3.16, 4.73)	4.72 (3.96, 5.21)	0.001
TG (mmol/L)	1.37 (1.09, 2.09)	1.47 (1.17,1.99)	0.165
preoperative Ca^2+^ (mmol/L)	2.69 (2.54,2.86)	2.89 (2.77,3.27)	<0.001*
postoperative P (mmol/L)	2.04 (1.72,2.32)	0.75 (0.61,0.94)	<0.001*
postoperative Ca^2+^ (mmol/L)	2.33 (2.05,2.46)	2.35 (2.16,2.69)	0.175
postoperative iPTH (pg/ml)	38.900 (19.60,61.00)	15.05 (8.15,28.94)	<0.001*
postoperative K^+^ (mmol/L)	5.51 (4.74, 6.12)	4.09 (3.80,4.40)	<0.001*
Decrease of iPTH (pg/ml)	2048.80 (1277.25, 2589.05)	228.75 (115.65, 482.50)	<0.001*

ACEI: angiotensin-converting enzyme inhibitor; Alb: serum albumin; ALP: alkaline phosphatase; ARB: angiotensin receptor blockers; BMI: body mass index; BUN: blood urea nitrogen; Ca^2+^: serum-corrected calcium; Chol: cholesterol; HCO3^-^: bicarbonate; iPTH: intact parathyroid hormone; K^+^: serum potassium; Mg: serum magnesium; P: serum phosphorus; PHPT: primary hyperparathyroidism; Scr: serum creatine; SHPT: Secondary hyperparathyroidism; TG: triglyceride;UA: uric acid; *: *p* < 0.05.

**Table 2.** Demographic features of hyperkalemic and nonhyperkalemic groups of postoperative patients with SHPT.

Characteristics	non-hyperkalemic group (*n* = 25)	hyperkalemic group (*n* = 28)	*p* value
Age(year)	50.00 ± 11.15	48.07 ± 13.53	0.576
Gender (*n*, male/female)	15/10	16/10	0.833
Current smoking (*n*, no/yes)	22/3	25/3	0.883
Dialysis duration year	7.44 ± 2.89	8.18 ± 3.04	0.371
BMI (kg/m^2^)	21.13 ± 2.34	21.66 ± 3.333	0.507
Interval time from dialysis to operation(h)	18.00 (14.50, 26.00)	18.00 (14.68, 20.75)	0.674
operating time (min)	134.80 ± 40.43	136.79 ± 43.62	0.865
preoperative Hb (g/L)	111.44 ± 16.38	109.89 ± 20.05	0.761
preoperative iPTH (pg/ml)	1824.92 ± 845.54	2212.57 ± 813.83	0.095
preoperative ALP (u/L)	401.00 (189.50, 872.00)	508.50 (344.50, 1062.75)	0.31
K^+^_base_ (mmol/L)	4.58 ± 0.59	5.28 ± 0.64	<0.001*
preoperative K^+^ (mmol/L)	4.24 (3.86, 4.65)	4.94 (4.58, 5.00)	<0.001*
preoperative HCO3^−^ (mmol/L)	23.12 ± 3.21	22.49 ± 2.40	0.418
preoperative BUN (mmol/L)	15.44 ± 4.24	17.94 ± 6.21	0.096
preoperative Scr (umol/L)	740.48 ± 201.00	701.51 ± 164.40	0.441
preoperative UA (umol/L)	311.69 ± 83.99	315.92 ± 96.36	0.866
preoperative Alb (g/L)	37.62 ± 4.72	37.13 ± 4.70	0.706
preoperative P (mmol/L)	2.15 ± 0.38	2.19 ± 0.36	0.728
preoperative Mg (mmol/L)	1.04 (0.95, 1.18)	1.07 (0.99, 1.24)	0.199
Chol (mmol/L)	3.65 (3.04, 4.86)	3.85 (3.41, 4.76)	0.669
TG (mmol/L)	1.26 (1.01, 2.21)	1.42 (1.14, 2.09)	0.643
preoperative Ca^2+^ (mmol/L)	2.59 ± 1.16	2.52 ± 0.68	0.781
postoperative P (mmol/L)	2.05 ± 0.52	2.02 ± 0.39	0.797
postoperative Ca^2+^ (mmol/L)	2.42 (2.12, 2.46)	2.27 (2.03, 2.48)	0.373
postoperative iPTH (pg/ml)	46.0 (18.02, 61.00)	32.15 (20.60, 61.13)	0.796
Decrease of iPTH (pg/ml)	1711.10 ± 798.38	2310.99 ± 826.81	0.066

Alb: serum albumin; ALP: alkaline phosphatase; BMI: body mass index; BUN: blood urea nitrogen; Ca^2+^: serum-corrected calcium; Chol: cholesterol; Hb: hemoglobin; HCO3^-^: bicarbonate; iPTH: intact parathyroid hormone; K^+^: serum potassium; K^+^base: baseline level of serum potassium; Mg: serum magnesium; P: serum phosphorus; Scr: serum creatine; SHPT: Secondary hyperparathyroidism; TG: triglyceride; UA: uric acid; *: *p* < 0.05.

**Figure 2.**
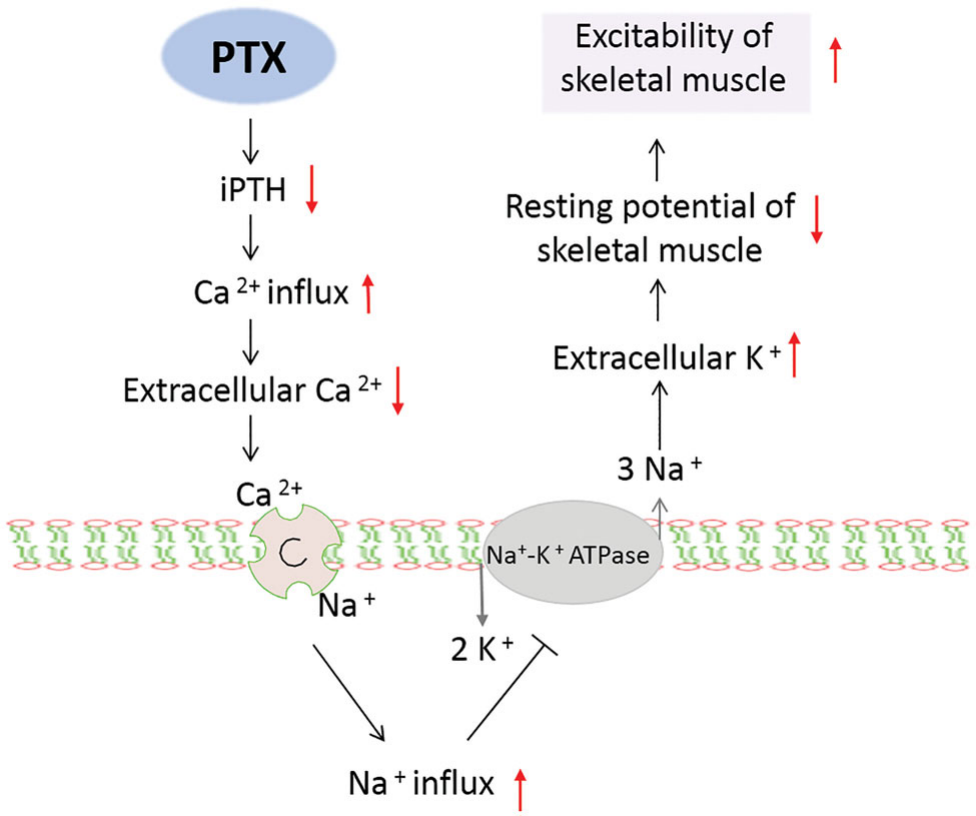
The possible interpretations of postoperative hyperkalemia undergoing parathyroidectomy in patients with secondary hyperparathyroidism.

Hyperkalemia after PTX in hemodialysis patients is common. Previous study has found that serum potassium could rise rapidly from 4.4 mmol/L to 6.2 mmol/L within operation time [1]. Rapidly rised severe hyperkalemia could lead to serious arrhythmia, even life-threatening [2]. Risk of hyperkalemia is more ominous in face of coexisting hypocalcemia (which is expected take place after PTX), so this issue is getting more and more attention in recent years. In our study, the incidence rate of hyperkalemia in hemodialysis patients after PTX is 52.8%, which is consistent with 25-80% occurrence reported in previous studies [3–7]. We speculate that the rapid decline of iPTH in a short time is an important reason for hyperkalemia after PTX. The possible interpretations are shown in Figure 2. Due to a rapid decline of iPTH after patients undergoing PTX, a large number of calcium ions influx into the bone make the level of calcium in extracellular fluid (ECF) in skeletal muscle cells (SMC) decrease [8]. An increased influx of sodium ions into SMC *via* membrane barrier action of sodium–calcium exchanger may influence the activation of Na/K ATPase pump which can promote efflux of sodium ion and influx of potassium. These result in increased level of potassium in ECF, reducing resting potential and increasing excitability of SMC [9–10]. A small sample study showed that ESRD patients with prior treatment with cinacalcet had a higher risk of hyperkalemia and hypocalcemia during and immediately after PTX [11]. However, we did not detect the significant association. Recent study has found preoperative serum potassium level <3.9 mmol/L would reduce the risk of developed potassium level >5.3 mmol/L in hemodialysis patients [12]. According to our study, reducing the K^+^_pre_ below 4.30 mmol/L is helpful to decrease the incidence of postoperative hyperkalemia.

Yun Zou *Department of Nephrology, The Third Affiliated Hospital of Soochow University, Changzhou, China* Liwei Zhang *Department of Infection Management, The Third Affiliated Hospital of Soochow University, Changzhou, China* Hua Zhou, Yan Yang, Min Yang and Jia Di *Department of Nephrology, The Third Affiliated Hospital of Soochow University, Changzhou, China* brightsea@163.com
